# Patient-Centered mHealth Intervention to Improve Self-Care in Patients With Chronic Heart Failure: Phase 1 Randomized Controlled Trial

**DOI:** 10.2196/55586

**Published:** 2025-01-15

**Authors:** Spyros Kitsiou, Ben S Gerber, Susan W Buchholz, Mayank M Kansal, Jiehuan Sun, Susan J Pressler

**Affiliations:** 1 University of Illinois Chicago Chicago, IL United States; 2 University of Massachusetts Chan Medical School Worcester, MA United States; 3 Michigan State University East Lansing, MI United States; 4 Indiana University Indianapolis, IN United States

**Keywords:** mHealth, app, digital health, telehealth, text messaging, smartphone, wearable electronic devices, heart failure, self-care, self-management, randomized controlled trial, cardiology, SMS

## Abstract

**Background:**

Heart failure (HF) is one of the most common causes of hospital readmission in the United States. These hospitalizations are often driven by insufficient self-care. Commercial mobile health (mHealth) technologies, such as consumer-grade apps and wearable devices, offer opportunities for improving HF self-care, but their efficacy remains largely underexplored.

**Objective:**

The objective of this study was to examine the feasibility, acceptability, safety, and preliminary efficacy of a patient-centered mHealth intervention (iCardia4HF) that integrates 3 consumer mHealth apps and devices (Heart Failure Health Storylines, Fitbit, and Withings) with a program of individually tailored SMS text messages to improve HF self-care.

**Methods:**

We conducted a phase 1 randomized controlled trial. Eligible patients had stage C HF, were aged ≥40 years, and had New York Heart Association (NYHA) class I, II, or III HF. Patients were randomly assigned to either iCardia4HF plus usual care or to usual care only and were observed for 8 weeks. Key feasibility measures were recruitment and retention rates. The primary efficacy outcome was change in HF self-care subscale scores (maintenance, symptom perception, and self-care management) at 8 weeks, assessed with the Self-Care Heart Failure Index (SCHFI; version 7.2). Key secondary outcomes were modifiable behaviors targeted by the intervention (health beliefs, self-efficacy, and HF knowledge), health status, and adherence to daily self-monitoring of 2 core vital signs (body weight and blood pressure).

**Results:**

A total of 27 patients were enrolled in the study and randomly assigned to iCardia4HF (n=13, 48%) or usual care (n=14, 52%). Of these 27 patients, 11 (41%) in the intervention group (iCardia4HF) and 14 (52%) in the usual care group started their assigned care and were included in the full analysis. Patients’ mean age was 56 (SD 8.3) years, 44% (11/25) were female, 92% (23/25) self-reported race as Black, 76% (19/25) had NYHA class II or III HF, and 60% (15/25) had HF with reduced left ventricular ejection fraction. Participant retention, completion of study visits, and adherence to using the mHealth apps and devices for daily self-monitoring were high (>80%). At 8 weeks, the mean group differences in changes in the SCHFI subscale scores favored the intervention over the control group: maintenance (Cohen *d*=0.19, 95% CI –0.65 to 1.02), symptom perception (Cohen *d*=0.33, 95% CI –0.51 to 1.17), and self-care management (Cohen *d*=0.25, 95% CI –0.55 to 1.04). The greatest improvements in terms of effect size were observed in self-efficacy (Cohen *d*=0.68) and health beliefs about medication adherence (Cohen *d*=0.63) and self-monitoring adherence (Cohen *d*=0.94). There were no adverse events due to the intervention.

**Conclusions:**

iCardia4HF was found to be feasible, acceptable, and safe. A larger trial with a longer follow-up duration is warranted to examine its efficacy among patients with HF.

**Trial Registration:**

ClinicalTrials.gov NCT03642275; https://clinicaltrials.gov/study/NCT03642275

## Introduction

### Heart Failure and Self-Care

Heart failure (HF) is a complex chronic condition with symptoms and signs that result from any structural or functional impairment of ventricular filling or ejection of blood [1]. HF affects >64 million people worldwide [2]. In the United States, approximately 6.7 million Americans aged >20 years have HF, and recent estimates indicate that by 2030, the prevalence of HF will increase to 8.5 million [3]. Black Americans have the highest incidence of HF among all racial and ethnic groups and approximately 45% greater risk of death or decline in functional status than White Americans [4]. The risk of developing HF is similar for women and men, but women are underrepresented in HF research [5].

Although considerable progress has been made over the years in both invasive and noninvasive treatments for the management of the disease [6], HF outcomes remain poor. HF is a leading cause of hospitalization and death among middle-aged and older adults in the United States [7,8]. Nearly 1 in 4 patients are readmitted within 30 days, and approximately 50% are readmitted within 6 months following an HF hospitalization [8-10]. Hospital readmissions result in significant costs to the health care system and are directly linked to poor health-related quality of life and depression [11,12]. The total cost for HF was US $30.7 billion in 2012 and is expected to reach US $69.7 billion by 2030 [13]. Therefore, attempts to decrease its societal and economic burden have become a major public health priority.

Self-care is considered essential for patients with HF, and therefore, improving self-care has increasingly become a major focus of multidisciplinary HF management programs and research worldwide [14]. HF self-care refers to behaviors and actions centered on 3 separate but linked concepts: *maintenance, symptom perception,* and *self-care management* [15]. The first concept, *maintenance*, refers to those behaviors that patients incorporate in their daily lives to maintain physiological stability and well-being (eg, taking medications as prescribed, following a heart-healthy diet, restricting sodium intake, and being physically active). *Symptom perception* involves daily self-monitoring and recognition of HF signs and symptoms (eg, sudden weight gain, increased blood pressure (BP), fatigue, shortness of breath, and chest pain) for early detection of deterioration. *Self-care management* refers to the strategies that one can use to respond to HF signs and symptoms when they occur (eg, adjusting diuretics, restricting fluid intake, adapting diet, and calling a care provider for guidance) to prevent hospital admission and increased mortality. A growing body of literature has shown that patients with HF who consistently engage in adequate HF self-care are more likely to be clinically stable, have better health-related quality of life, and have better event-free survival [16-20]. However, self-care in patients with HF is frequently poor, especially among racial and ethnic minority patients due to socioeconomic factors that account for delays in seeking treatment for worsening symptoms, inadequate access to health care, noncompliance with follow-up appointments, and poor adherence to recommended treatments [1,4]. Even patients recently discharged from the hospital due to acute decompensated HF (ADHF), where they presumably received patient education about HF, demonstrate low rates of adherence to basic HF self-care behaviors, such as taking HF medications as prescribed, following a low-sodium diet, and self-monitoring for HF signs and symptoms [21]. Multidisciplinary HF disease management programs that involve hospital or clinic intervention (eg, regular follow-up visits and patient education) by a multidisciplinary team of cardiologists, advanced practice nurses, pharmacists, dieticians, or other HF specialists have been successful at reducing mortality and HF-related hospitalizations. However, such programs are not available to all patients and have limited reach due to limited health care resources and accessibility constraints [22]. Therefore, interventions have evolved toward a proactive, real-time technological model to better monitor and assist patients with HF self-care and management of symptoms at home. These interventions range from structured telephone support and noninvasive home telemonitoring [23,24] to remote patient monitoring (RPM) with implantable devices [25,26] and, more recently, the use of mobile health (mHealth) technologies for patient education [27] and HF self-care support [28-31].

### mHealth Interventions

mHealth, which is defined as the use of mobile computing and communication technologies in health care and public health, is a rapidly expanding area within biomedical and health informatics with great potential for developing and delivering patient-centered behavioral interventions to improve self-care and chronic disease management [32-34]. Τhe term *patient-centered* is used in this paper to refer to interventions that focus explicitly on the patient and in which the content of the intervention is selected or tailored to address the patient’s salient characteristics (eg, health beliefs, knowledge, confidence, cultural background, and preferences) [35]. With the uptake of smartphone ownership among adults in the United States [36], there is a growing opportunity to capitalize on the use of mHealth apps, wearable sensor devices, and other connected health technologies to develop patient-centered HF self-care interventions that are more accessible and scalable. As patients carry their smartphones with them wherever they go, patient-centered mHealth interventions can fit more seamlessly into their daily routine and lifestyle to support their needs.

Consumer-grade mHealth technologies hold promise for engaging patients in better HF self-care and delivering patient-centered intervention, but their efficacy remains underexplored. A recent systematic review and meta-analysis found that mHealth interventions for patients with HF reduced the risk of all-cause mortality and HF hospitalizations [29]. However, results were mainly driven by studies that implemented mHealth as part of a larger RPM system that focused on daily transmission of patient-generated health data to a clinical care team for review and delivery of actionable feedback. Fewer studies tested the effects of stand-alone mHealth interventions (without RPM) focused on improving self-care, and virtually no studies tested the efficacy of commercially available mHealth apps, despite their increasing availability and uptake [28]. Furthermore, none of the interventions were tailored to the patients’ characteristics or needs, which is often one of the reasons for low engagement with and abandonment of mHealth apps and devices [37,38].

### Study Objectives

To address this important knowledge gap, our team developed and tested a patient-centered HF self-care intervention (iCardia4HF) that integrates 3 commercial mHealth apps and devices (MyApps) with a program of individually tailored SMS text messages (Text4HF) to improve self-care in patients with HF. Tailoring of the text messages was guided by participants’ health beliefs, knowledge, and confidence in performing HF self-care. The intervention was delivered through an innovative digital health platform (iCardia) [39], which allows for remote collection of patient-generated health data from the devices and apps and delivery of the tailored text messages. The objective of this study was to test the feasibility, acceptability, and safety of the iCardia4HF intervention and to determine the effects of the intervention on HF self-care (primary outcome) and other health outcomes over 8 weeks, compared with usual care.

### Theoretical Basis and Development of the Intervention

Details regarding the structure, content, and delivery of the intervention are provided in the Methods section. Here, we briefly describe the theoretical basis and development of the intervention.

**Figure 1:**
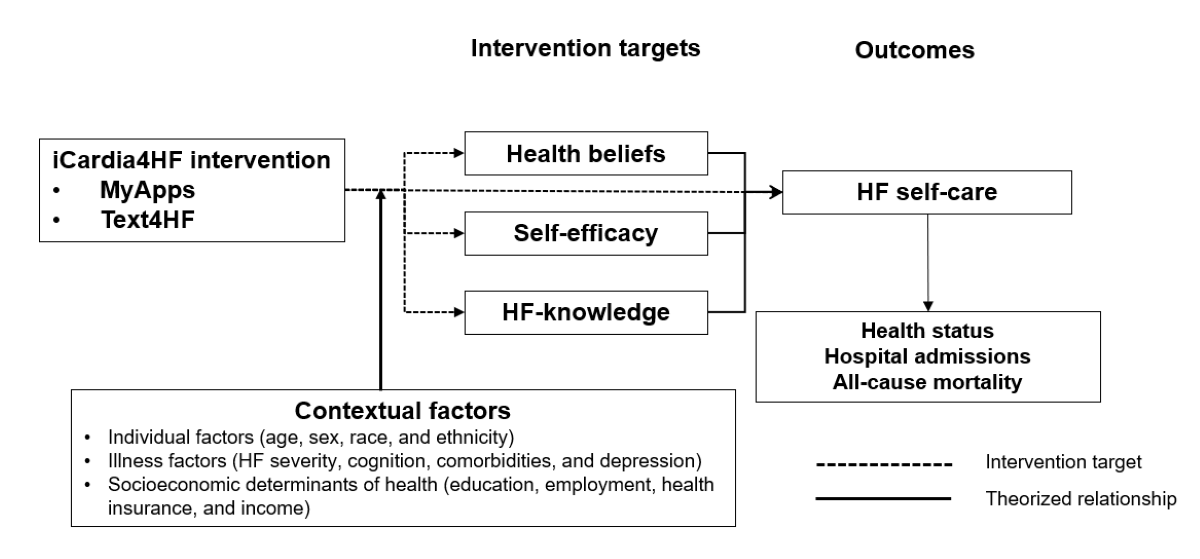
Conceptual framework providing the basis for the intervention. HF: heart failure.

Figure 1 presents the conceptual framework that formed the basis for the iCardia4HF intervention. The intervention is based on the situation-specific theory of HF self-care [15] and the Health Belief Model [40]. In the situation-specific theory, HF self-care is defined as a naturalistic decision-making process involving routine behaviors and actions that maintain physiological stability *(maintenance)*, facilitate self-monitoring and identification of HF symptoms *(symptom perception)*, and direct the patient’s response to those symptoms *(self-care management)*. The Health Belief Model proposes that individuals’ performance of a specific self-care behavior (eg, medication adherence or following a sodium-restricted diet) is determined by their beliefs regarding susceptibility to the disease, seriousness of the disorder, and perceived benefits and barriers of performing that self-care behavior. In addition to health beliefs, HF self-care decisions are influenced by knowledge about the disease (HF knowledge) [41,42] and confidence (self-efficacy) in performing HF self-care [43,44]. Confounding HF self-care decisions are contextual factors [21,45] which include the individual characteristics of a person (eg, age, gender, race, and ethnicity), the illness-related factors (eg, HF severity, comorbidities, and depression), and social determinants of health (eg, education, employment, health insurance, and income).

The iCardia4HF intervention was developed iteratively following a user-centered design process [46], informed by the needs of key stakeholders (patients, clinicians, and researchers) and our team’s expertise in mHealth and chronic disease management. For the MyApps component of the intervention, we selected 3 popular consumer-grade mHealth apps (Heart Failure Health Storylines [Self-Care Catalysts Inc], Withings Health Mate [Withings], and Fitbit [Google LLC]) and 3 connected health devices (Withings Body Cardio Scale, Withings Blood Pressure cuff, and Fitbit Charge 2 activity tracker). These apps and devices incorporate core functionalities and electronic tools (eg, adherence reminders, vital signs monitoring, symptoms tracking, and medication tracking) that support the main aspects of HF self-care (ie, maintenance, symptom perception, and self-care management). For the selection of the mHealth apps and connected health devices that make up the MyApps intervention component, we relied on a systematic review of 30 HF self-care apps [28] and a cross-sectional survey study of 100 patients with HF [36] in which we assessed attitudes and perceptions toward mHealth technologies, including current use and preferences for mHealth apps and devices. We also relied on cumulative experience gained from other interventional studies we have conducted in people with cardiovascular disease and other chronic diseases, in which we used Fitbit and Withings devices for vital signs monitoring and promotion of physical activity and healthy lifestyle behaviors [47-50]. Fitbit devices are increasingly used in research [51] and have been noted to be valid, reliable, and user-friendly in monitoring physical activity [52,53]. Similarly, Withings scales and BP monitors are accurate and have received 510 (k) clearance by the US Food and Drug Administration. By using these mHealth apps and devices regularly, patients with HF can become more aware of how their bodies work and what is normal, identify health changes that may need medical attention, and stay motivated while making healthier lifestyle choices.

The Text4HF component of the intervention consists of individually tailored text messages that aim to promote HF self-care adherence by targeting patients’ health beliefs about HF self-care (ie, perceived barriers and benefits to prescribed medications, sodium-restricted diet, self-monitoring of HF symptoms, and daily physical activity), including HF knowledge and self-efficacy. For the development of the Text4HF intervention component, we relied on and built upon the work of Dr Susan Pressler, an internationally recognized expert in health behavior interventions for patients with HF. First, we translated the original messages that were developed and tested in the *Heart Messages* [54] study and in the *Promoting Understanding and Management through Partnering: U and Your Physician* [55] study, into SMS text messages. Then, we developed additional text messages to strengthen the intervention. Finally, we refined the text messages based on clinical guidelines for the management of HF [56] and stakeholder input to ensure accuracy, readability, and cultural relevance [57].

## Methods

### Study Design

iCardia4HF was a phase 1 (feasibility and pilot) randomized controlled trial [58] with 2 arms (intervention and control), allocation concealment, and masking of outcome assessors to group assignment. The intervention duration was 8 weeks. Patient assessments were performed in person at baseline, 4 weeks (30 days), and 8 weeks (56-60 days). The primary outcome of interest was HF self-care. The duration of the intervention and selection of follow-up visits for the assessment of HF self-care and intervention target variables were chosen based on guidelines for feasibility studies and because improvements in HF self-care and other measures relevant to this study have occurred at similar times in other studies [55,58-60]. The trial was conducted between January 2019 and February 2020.

### Population and Setting

Patients were recruited from the hospital and outpatient HF clinic of the University of Illinois Hospital and Health Sciences System (UI Health) in Chicago, Illinois. Inclusion criteria were (1) chronic HF (stage C); (2) New York Heart Association (NYHA) class I, II, or III; (3) aged ≥40 years; (4) ability to speak and read in English; (5) smartphone ownership; and (6) living within a 48-km radius from UI Health. Exclusion criteria were (1) on a waiting list for an implanted ventricular assist device or heart transplant; (2) advanced renal disease (hemodialysis or creatinine >4.0 mg/dL) (3) end-stage HF; (4) active cancer; (5) not able to perform self-care; (6) living in a setting other than home (eg, nursing home); (7) hospice candidate; (8) Montreal Cognitive Assessment score <22 [61]; and (9) major cognitive impairment (eg, dementia). Originally, the age eligibility criterion was set to ≥50 years in this study, but due to the large number of African American patients served by the UI Health system and the fact that on average HF occurs in Black patients 10 to 15 years earlier, we modified our study protocol to include patients aged ≥40 years [62]. On the basis of left ventricular ejection fraction (EF), HF is classified into 3 EF categories: HF with reduced EF (HFrEF), mildly reduced EF (HFmEF), and preserved EF (HFpEF), according to the EF ranges ≤40%, 41% to 49%, and ≥50%, respectively. Preserved EF now represents >50% of HF cases and can have outcomes as poor as those associated with HF with reduced EF and mildly reduced EF [1]. Therefore, we included patients from all 3 EF categories.

### Sample Size

The primary purpose of a phase 1 study is to determine the feasibility of recruitment, retention, and intervention delivery and to collect preliminary data on the primary outcome measure to inform the design and sample size calculation for a phase 2 trial. Hence, an efficacy-based power analysis was beyond the scope of this feasibility study [63]. During the planning stages of the trial (grant proposal submission) and in the original protocol that we published in clinicaltrials.gov before the start of recruitment, we aimed at enrolling 40 patients (an average of 4 patients per month over a 10-month recruitment period), using the general rule of thumb of having 15 to 20 patients per group to obtain an estimate of the variance for the primary outcome. However, as the study progressed and we collected recruitment data, we revised the original accrual plan, taking into consideration the eligibility fraction (proportion of potential participants who undergo screening and are eligible to enroll), the enrollment fraction (proportion of people who are eligible for participation and who actually enroll for the trial), and the funding period for the study (12 months). Consistent with recommendations that a sample size of 12 participants per group is suitable for a phase 1 trial [64], we adjusted the anticipated enrollment in the study to 28 participants, accounting for an expected attrition rate of 15%. The revised justifications for this sample size were based on rationale about feasibility and precision about the mean and variance, as described in the study by Julious [64]. Specifically, we considered the degrees of freedom required to ensure the 95th percentile for the variance has 50% power.

### Screening and Recruitment Process

Patient screening and recruitment were performed in 2 stages. Stage 1 involved querying the hospital electronic medical record to identify and screen potentially eligible patients who were either admitted to the hospital due to HF or had an upcoming appointment at the outpatient HF clinic for follow-up. Patients found eligible on initial screening were approached in person by a study researcher who briefly described the study, invited participation, and further assessed patients for eligibility (stage 2). Eligible patients agreeing to participate provided written informed consent, and Health Insurance Portability and Accountability Act (HIPAA) authorization and were scheduled for baseline assessment and study orientation.

### Randomization and Allocation Concealment

After completion of baseline assessment, patients were randomly assigned 1:1 to the intervention or the control group. For the randomization, we used the biased-coin minimization method by Pocock and Simon [65] with base probability set to 0.7 to achieve balance of important clinical covariates (age, biological sex, and NYHA functional class) between the 2 study groups. Randomization was performed centrally by a designated staff person using QMinim [66], password-protected web-based software hosted in a secure server at the University of Illinois Chicago. The person responsible for the randomization was not involved in patient recruitment and enrollment and did not have the ability to influence the execution of the randomization procedure. Investigators enrolling participants did not have access to the system or knowledge of the minimization algorithm and therefore could not foresee assignments.

### iCardia4HF Intervention Group

Patients in the intervention group received the MyApp and Text4HF components of the iCardia4HF intervention, in addition to usual care. In the subsequent sections, we describe the content, structure, and delivery of the 2 intervention components.

#### Consumer mHealth Apps and Devices (MyApps)

##### Overview

MyApps consisted of 3 popular consumer-grade mHealth apps (Heart Failure Health Storylines [version 7.17], Withings Health Mate [version 4.1.1], and Fitbit [version 2.86]), and 3 connected health devices (Withings Body Cardio Scale [version 1751], Withings Blood Pressure cuff [version BP-801], and Fitbit Charge 2 activity tracker [version 22.58.0]). The mobile apps and devices interface with each other, offering patients an integrated set of self-monitoring and behavior change tools that support the 3 core elements of HF self-care (maintenance, symptom perception, and self-care management) [15]. There were no major changes or upgrades to the apps and devices during the study affecting their content or features. Also, no adaptations or changes were made to the consumer apps or devices used in the study as part of the intervention. Each app specializes in different preventative and healthy lifestyle behaviors, and collectively, the 3 apps and devices supported the following HF self-care activities:

##### Medication Adherence

Heart Failure Health Storylines has a medication tracking feature that allows patients to add the medications they are taking and schedule reminders in the form of push notifications for each medication. When a medication reminder is triggered on the phone, patients are prompted to respond whether they took their medication or not, and thus maintain a digital diary of medication adherence on their phone.

##### Vital Signs Monitoring

HF self-care requires daily monitoring of weight to check for weight gain caused by increased fluid [1]. It also requires lowering BP to make it easier for the heart to pump blood [1]. Monitoring of weight and body composition (fat mass, muscle mass, water mass, bone mass, and BMI) is captured by the Withings Body Cardio scale. BP, including heart rate, is captured by the Withings Blood Pressure monitor. Both devices interface with the Withings Health Mate app via Bluetooth and automatically transmit captured data whenever a new measurement is taken. For weight and body composition, the app provides patients with visual line graphs along with textual feedback to notify them of any fluctuations over time (eg, gaining weight +2.2kgs since last measurement or over a selected period). Similarly, BP measurements (systolic and diastolic) are presented in the app in tabular format and also as a line graph, using a color-coding scheme to classify each measure as normal (<120/80 mm Hg), elevated (120−129/<80 mm Hg), high BP or stage 1 hypertension (130-139/80-89 mm Hg), high BP or stage 2 hypertension (140-179/90-119 mm Hg), or hypertensive crisis (≥180/≥120 mm Hg), according to clinical guidelines [67]. In case BP>180/110 mm Hg, the app advises the patient to seek emergency care right away.

##### HF Symptom Monitoring

In addition to taking medications and tracking weight and BP, HF self-care involves monitoring HF symptoms. The Heart Failure Health Storylines app has a feature that allows patients to record the presence and severity of HF symptoms over time, using a 10-point Likert scale. Symptoms are timestamped and displayed in a weekly calendar format using a color-coded scheme. Symptom questions programmed in the app at baseline included chest pressure; coughing; difficulty in sleeping; swelling in the abdomen; swelling of legs, feet, and ankle; shortness of breath when lying down or at rest; shortness of breath with activity; and fatigue. Patients were asked to perform symptom tracking daily.

##### Sodium-Restricted Diet

Patients with HF typically need to restrict sodium intake to avoid fluid retention and episodes of ADHF. The Heart Failure Health Storylines app provides patients with generic information about heart healthy eating and guidelines for lowering sodium intake.

##### Physical Activity

The Fitbit app and Charge 2 activity tracker provide patients with several tools that support daily self-monitoring of steps, intensity of physical activity, heart rate, and sleep. Also, Fitbit incorporates a variety of behavior change tools, such as goal setting, activity visualization with feedback, rewarding messages when step goals are met, and prompts to move when sedentary (<250 steps) for >50 minutes.

All patient-generated health data captured by the connected health devices and mHealth apps are presented in the “My Storylines” dashboard of the Heart Failure Health Storylines app as visual graphs in a weekly calendar format with color-coded schemes. This helps patients identify preclinical measures of ADHF between time periods, correlate lifestyle behaviors with changes in their health, and modify their behavior accordingly.

We used a goal-based approach to promote self-care via the mHealth tools described earlier in concurrence with clinical guidelines for patients with HF. The primary goals communicated to patients at baseline were (1) daily self-monitoring of weight and BP each morning before breakfast and medications, (2) self-recording of HF symptoms, (3) recording of medication taking by replying (yes or no) to the push notifications, (4) gradual increase of physical activity to reach 20 to 30 minutes of moderate-to-vigorous physical activity (MVPA) per day most days of the week, and (5) following a low-sodium diet (2000-3000 mg of sodium/day).

#### Individually Tailored Text Messages (Text4HF)

Text4HF is a program of individually tailored text messages targeting patients’ health beliefs (perceived barriers and benefits), self-efficacy, and knowledge about HF self-care. Patients in the intervention group received 4 text messages per week, at a day and time of their preference, via SMS. Tailoring of the text messages was guided by patients’ responses to valid and reliable assessments of intervention target variables assessed at baseline and after 4 weeks . Items on the Health Belief scale are divided into benefit and barrier questions regarding adherence to prescribed medications, sodium-restricted diets, and self-monitoring of weight and HF symptoms. Each item on the scale received a 5-point score ranging from 1 (strongly disagree) to 5 (strongly agree). Participants who scored ≥3 on a barrier question or ≤3 on a benefit question received text messages tailored to that specific barrier or benefit item (Table 1). If a participant scored >3 on a benefit question or <3 on a barrier question, then a message in relation to those items was not sent because it was presumed that the patient already understood the barriers or benefits identified in that question. A similar approach was followed for the other 2 intervention target variables: self-efficacy and HF knowledge. For example, patients who gave an incorrect answer on an item of the Dutch Heart Failure Knowledge Scale received a patient education message in relation to that item.

**Table 1:** Sample tailored text messages based on responses to validated questions.

Intervention target	Question	Rating scale	Decision algorithm	Example text message
Medication adherence	Benefit question: if I take my water pills, I will lower my chance of being in the hospital.	5-point Likert (1 is strongly disagree and 5 is strongly agree)	If answer ≤3, then send text message	Taking water pills as prescribed can help remove extra water from the body and lessen the risk of getting hospitalized
Medication adherence	Barrier question: taking water pills makes it hard to go away from home.	5-point Likert (1 is strongly disagree and 5 is strongly agree)	If answer ≥3, then send text message	If taking water pills makes it hard to go away from home, one option is to take it several hours before you plan to go out or wait until after you return home to take it.
Diet adherence	Barrier question: food does not taste good on the low salt diet.	5-point Likert (1 is strongly disagree and 5 is strongly agree)	If answer ≥3, then send text message	You can flavor your food without using salt. You can use seasonings like pepper, lemon juice, garlic, onion powder, and basil.
Self-monitoring adherence	Barrier question: weighing myself is unpleasant.	5-point Likert (1 is strongly disagree and 5 is strongly agree)	If answer ≥3, then send text message	Although weighing yourself daily might be unpleasant, it is important to do so because it can help you identify fluid buildup in your body.
Self-care self-efficacy	How confident are you that you can keep yourself stable and free of symptoms	5-point Likert (1 is not confident and 5 is extremely confident)	If answer ≤3, then send text message	(Message 1) Following your doctor’s advice and taking your medications as directed are important steps to managing your condition. (Message 2) Tell your doctor right away if you gain 3 or more pounds in a day or feel swelling in your feet, ankles, and other parts of your body or if it is hard to breathe.
Heart failure knowledge	What is the best thing to do in case of increased shortness of breath or swollen legs?	Multiple choice answer	If answer is other than “call the doctor or nurse,” then send a text message	Call your health care provider if you feel any of the following: shortness of breath when lying flat or standing and swelling in your legs or stomach.

### Control Group

Patients allocated to the control group received usual care provided through the outpatient HF clinic and primary care. Guideline-directed medical therapy, included initial and serial evaluation of HF (eg, physical examination, diagnostic tests, biomarkers, vital signs, symptoms, left ventricular EF [LVEF], volume status, weight, jugular venous pressure, and presence of edema); pharmacological treatment with diuretics, angiotensin-converting enzyme inhibitors, Angiotensin II receptor blockers, β blockers, and aldosterone receptor antagonists; nonpharmacological intervention (eg, patient self-care education and referral to cardiac rehabilitation); care coordination, and regular follow-up appointments with a cardiologist and Advanced Practice Registered Nurse at the Heart Failure Clinic 7 days postdischarge and every 2 to 3 months thereafter for further assessment and treatment.

### Study Procedures

All study procedures were carried out in private examination rooms by certified key research personnel with training on human subjects research and HIPAA. After completion of baseline assessment and randomization, patients allocated to the intervention group received instructions and in-person training on how to use the mHealth apps and devices using the teach-back method. A research assistant downloaded the intervention apps on each participant’s smartphone and then used a deidentified email address created specifically for this study to sign each patient into the intervention apps and pair the apps with their respective device via Bluetooth. Next, participants were shown how to use each app, sync the study devices with their personal smartphones, and view their data using the apps. Participants were introduced to the “position control” technology implemented in the Withings devices and were shown how to use the on-screen visual indicators to center their body on the scale and properly place the BP cuff on their arm to ensure consistent and accurate measurements. As part of our study protocol and in accordance with clinical guidelines about HF self-care, patients were advised to use the mHealth devices and apps to record their weight, BP, and symptoms every morning after going to the bathroom and before eating breakfast and taking their medications. Daily reminders in the form of push notifications for medications, symptom tracking, and monitoring of weight and BP were programmed in each app according to patients’ daily routines and preferences. To reduce complexity and participant burden, medication reminders were set up for 1 medication only (angiotensin-converting enzyme inhibitor, loop diuretic, or β-blocker) based on the documented regimen in the patient’s electronic medical record and discussion with the patient at baseline. Patients were trained how to edit existing medications or add new ones and how to change the time of medication reminders if needed. At the end of the training session, participants were provided with an end user’s manual that contained instructions on how to use each app and device and a copy of their study email and password for logging into the apps. Also, they were provided with a phone number to contact our team in case they experienced technical issues or had study-related questions. During training we emphasized to patients that the intervention was not a substitute for usual care and did not serve as a RPM system for identifying potential exacerbations or reporting medical emergencies.

The iCardia platform [39] was used to remotely collect all patient-generated health data captured by the mHealth apps and devices and to send the intervention text messages. A trained research assistant (interventionist) programmed the text messages in iCardia after completion of baseline and 4-week visits. iCardia is a secure, web-based system that is hosted on a HIPAA-compliant server at the University of Illinois Chicago and has been validated through multiple trials [47,49,50,68-71].

### Masking of Outcome Assessors

By design, group assignments were identifiable to study participants, but outcome assessors and care providers treating patients were masked to group assignment. Outcome assessors worked in different office space than other study researchers who were aware of the group allocation. Before outcome assessment, patients were asked not to reveal to outcome assessors which group they were assigned to and were also asked to remove the Fitbit from their wrist to conceal their allocation. All outcome assessors were trained and continuously monitored.

### Study Measures and Data Collection Schedule

#### Overview

Study participants were observed for 8 weeks. Table 2 lists all the measures and data collection schedule by outcome of interest. Once consent was obtained, participants provided basic demographic information (age, sex, race, ethnicity, employment status, marital status, living arrangement, education, and health insurance), and completed the Montreal Cognitive Assessment test [61] and Patient Health Questionnaire [72]. Clinical data, such as severity of HF (eg, LVEF and NYHA), primary cause of HF, comorbidities, BP, and anthropometrics (height, weight, and BMI), were retrieved from the electronic medical record at baseline. Study visits and assessments occurred in person at baseline, 4 weeks, and 8 weeks. Data from the mobile apps and devices were collected daily during the 8-week follow-up period.

**Table 2:** List of measures and data collection schedule.

Outcomes	Measures	Baseline	4 weeks	8 weeks
Demographics	Age, sex, race, ethnicity, marital status, employment status, education, financial status, living arrangement, and health insurance	✓		
Clinical characteristics	LVEF^a^, NYHA^b^ class, BP^c^, weight, height, and BMI.	✓		
Cognitive function	Montreal Cognitive Assessment test [61]	✓		
Feasibility (recruitment and retention)	Number of patients screened, number of eligible and ineligible patients, reasons for exclusion, recruitment rate, and retention rate	Monitored throughout the study	Monitored throughout the study	Monitored throughout the study
Acceptability	Technology acceptance questionnaire (perceived usefulness, ease of use, user satisfaction, confirmation of initial expectations, and intention to continue using the intervention apps and devices)			✓
HF^d^ self-care (primary)	Self-Care Heart Failure Index (version 7.2)	✓	✓	✓
Self-efficacy	Self-Care Self-Efficacy scale	✓	✓	✓
Health beliefs	Beliefs about Medication Adherence Scale, Beliefs about Diet Adherence Scale, Beliefs about Self-Monitoring Adherence Scale	✓	✓	✓
HF-knowledge	Dutch Heart Failure Knowledge Scale	✓	✓	✓
Health status	Kansas City Cardiomyopathy Questionnaire	✓	✓	✓
Health care encounters	Hospitalizations and ER^e^ visits were identified and recorded using the electronic medical record system and patient interviews.	✓	✓	✓
Mortality	Death certificate or other report in the electronic medical record system	✓	✓	✓
Self-monitoring adherence	Daily weighing and BP monitoring—timestamped data from the Withings Body Cardio Scale and BP cuff	Monitored throughout the study	Monitored throughout the study	Monitored throughout the study
Steps	Mean number of daily steps were measured with the Fitbit Charge 2 activity tracker.	Monitored throughout the study	Monitored throughout the study	Monitored throughout the study
MVPA^f^ minutes	Mean number of MVPA minutes/day were measured with the Fitbit Charge 2.	Monitored throughout the study	Monitored throughout the study	Monitored throughout the study

^a^LVEF: left ventricular ejection fraction.

^b^NYHA: New York Heart Association.

^c^BP: blood pressure.

^d^HF: heart failure.

^e^ER: emergency room.

^f^MVPA: moderate-to-vigorous physical activity.

#### Feasibility Outcome Measures

Feasibility was assessed in the domains of patient recruitment and retention during the 8-week follow-up period. Feasibility measures assessed throughout the study included the following: (1) number of patients screened, (2) number of eligible patients, (3) reasons for ineligibility, (4) recruitment rate (mean number of patients recruited per month), and (5) retention rate (percentage of randomized participants who completed the study).

#### Primary Efficacy Outcome Measure

The primary efficacy outcome measure was self-reported HF self-care assessed with the Self-Care Heart Failure Index (SCHFI; version 7.2) [15] at baseline, 4 weeks, and 8 weeks (Table 2). SCHFI is a validated instrument that contains the following three subscales: (1) Self-care Maintenance (10-items), (2) Symptom Perception (9-items), and (3) Self-care management (10-items). Each subscale is scored separately. Response choices for all items in each subscale are summed and standardized to achieve a possible score of 0 to 100. Higher scores indicate better HF self-care. Scores <70 indicate insufficient self-care. The minimal clinically important change in SCHFI is one-half SD, or an 8-point difference in the standardized score of each subscale [73].

#### Secondary Outcome Measures

Secondary outcome measures (Table 2) included self-efficacy, health beliefs, HF-knowledge, health status, adherence to daily self-monitoring of weight and BP, physical activity, acceptance of the intervention, and adverse events.

#### Self-Efficacy

Confidence in performing routine HF self-care behaviors was assessed with the Self-Care Self-Efficacy questionnaire that has 10 items [15,74]. Similar to SCHFI, items in the Self-Care Self-Efficacy scale are summed and standardized to achieve a possible score of 0 to 100. Higher scores indicate greater confidence in HF self-care. Scores <70 indicate inadequate self-efficacy. The minimal clinically important change is one-half SD, or an 8-point difference in the standardized score [73].

#### Health Beliefs

Perceived benefits and barriers about HF self-care were assessed using three validated scales: (1) Beliefs about Medication Adherence Scale (12 items) [75], (2) Beliefs about Diet Adherence Scale (12 items) [75], and (3) Beliefs about Self-Monitoring Adherence Scale (12 items) [76].

#### HF Knowledge

The Dutch Heart Failure Knowledge Scale was used to measure general knowledge about HF, knowledge about HF treatment (including diet and fluid restriction) and symptoms, and symptom recognition [77]. The Dutch Heart Failure Knowledge Scale is a 15-item questionnaire that has a minimum score of 0 (no knowledge) and a maximum score of 15 points (optimal knowledge).

#### Health Status

The short version of the Kansas City Cardiomyopathy Questionnaire 12-item (KCCQ-12) was used to assess health status [78]. KCCQ-12 is a validated health status measure for patients with HF that contains 4 subscales: physical limitation, symptom frequency, quality of life, and social limitation. Each subscale provides an individual score from 0 to 100, with 0 denoting the worst and 100 the best possible health status. The mean of the 4 subdomain scores is presented as a summary score, with differences of ≥5 points considered clinically important [79].

#### Self-Monitoring Adherence

Daily adherence to self-monitoring of weight and BP was assessed using timestamped data automatically transmitted from the Withings devices and app to the iCardia server. Adherence was defined as the percentage of days patients used the Withings Body Composition scale and BP cuff along with the Health Mate app to measure their weight and BP over the 8-week follow-up period.

#### Physical Activity (Steps and MVPA)

Daily steps and MVPA were assessed with the Fitbit Charge 2 activity tracker that participants received as part of the intervention during 8 weeks. Participants were asked to wear the Fitbit daily for ≥10 hours (600 minutes) every day during waking hours and gradually increase their physical activity over time. A valid day consisted of 10 hours of wear time and a valid week consisted of at least 4 valid days. Daily Fitbit wear time was calculated by iCardia based on the collected heart rate data using a previously validated method [47,80].

#### Adverse Events

As part of our safety protocol and monitoring of adverse events, we recorded all hospitalizations, emergency room visits, and deaths that occurred during the follow-up period of our trial. At each study visit, a trained research assistant reviewed the electronic medical record of each patient (encounters and provider notes) and asked them for possible hospitalizations that occurred at UI Health or elsewhere, including adverse events. The study physicians adjudicated the encounters and events per our study safety protocol.

#### Intervention Acceptability

Exit interviews were conducted at 8 weeks (postintervention) with patients allocated to the iCardia4HF group to assess *acceptance* and elicit their feedback about the intervention. Measures of technology acceptance—adapted to the context of this study from the Technology Acceptance Model [81] and the Expectation-Confirmation Model of continued information technology use [82,83]—were assessed using a 3-point Likert scale and included the following: (1) perceived usefulness (7 items), (2) ease of use (5 items), (3) user satisfaction (3 items), (4) confirmation of initial expectations (3 items), and (5) intention to continue using the mobile apps and devices of the iCardia4HF intervention after the end of the study (3 items).

### Statistical Analysis and Interpretation

All analyses were conducted using an intent-to-treat approach (ie, participants were analyzed according to their assigned group) independent of the actual intervention dose received. All variables were checked for errant values. Descriptive statistics were computed for all items and scale scores. Distributions were examined for nonnormality and transformed if necessary. To deal with missing data, we used a multiple imputation approach [84]. Imputation of the missing values for each outcome was performed by treatment group, age, gender, BMI, NYHA, LVEF, Patient Health Questionnaire-9, and Montreal Cognitive Assessment to create 20 imputed datasets using the mice package [85] in R software (version 4.2.1; R Core Team) [86]. Analyses of between-treatment differences in primary and secondary outcomes were performed using a 2-sample *t* test to assess the intervention effect. Our approach to data reporting and interpretation regarding the intervention effects on HF self-care and secondary outcomes was focused on the direction and magnitude of treatment effect estimates, as well as the uncertainty behind each estimate (95% CI), and not on *P* values [87].

Daily weighing and BP self-monitoring adherence in the intervention group was measured by week (as the percentage of protocol-required measurements that were completed during the follow-up period), after adjusting for days outside of the hospital. For the Fitbit physical activity data, we first calculated the mean number of daily steps and daily MVPA minutes per week using all valid days for each participant. A valid day was one that had ≥600 minutes of wear time. We only included weeks that had ≥3 valid days. We also calculated the mean daily sedentary minutes and Fitbit wear time per day. To study the trajectories of the mean number of daily steps per week for the intervention group, we used linear mixed-effects models [88], where the continuous week variable was included as a fixed effects and a random intercept was included to account for the intrasubject correlations. We adopted the same analysis strategy to study the trajectories of the mean daily MVPA minutes, mean daily sedentary minutes, mean daily Fitbit wear-time, and mean daily weight and BP monitoring adherence for the intervention group. We used the Non-Linear Mixed Effects package [89] to fit the linear mixed-effects model in R.

### Ethical Considerations

The study protocol was approved by the university’s institutional review board, registered on ClinicalTrials.gov (NCT03642275), and reported in accordance with the CONSORT-eHEALTH checklist (Consolidated Standards of Reporting Trials of Electronic and Mobile Health Applications and Online Telehealth; Multimedia Appendix 1). As compensation for their time and participation in the study, patients received US $25 for completing the baseline assessment, US $30 for completing the 4-week study visit, and US $30 for completing the 8-week study visit, which amounts to a total of US $85. They were also able to keep the apps and devices after completion of the study. In addition, they were provided with free parking and transportation reimbursement from and to University of Illinois Chicago and UI Health for study visits as needed.

## Results

### Description of the Population Sample

**Figure 2:**
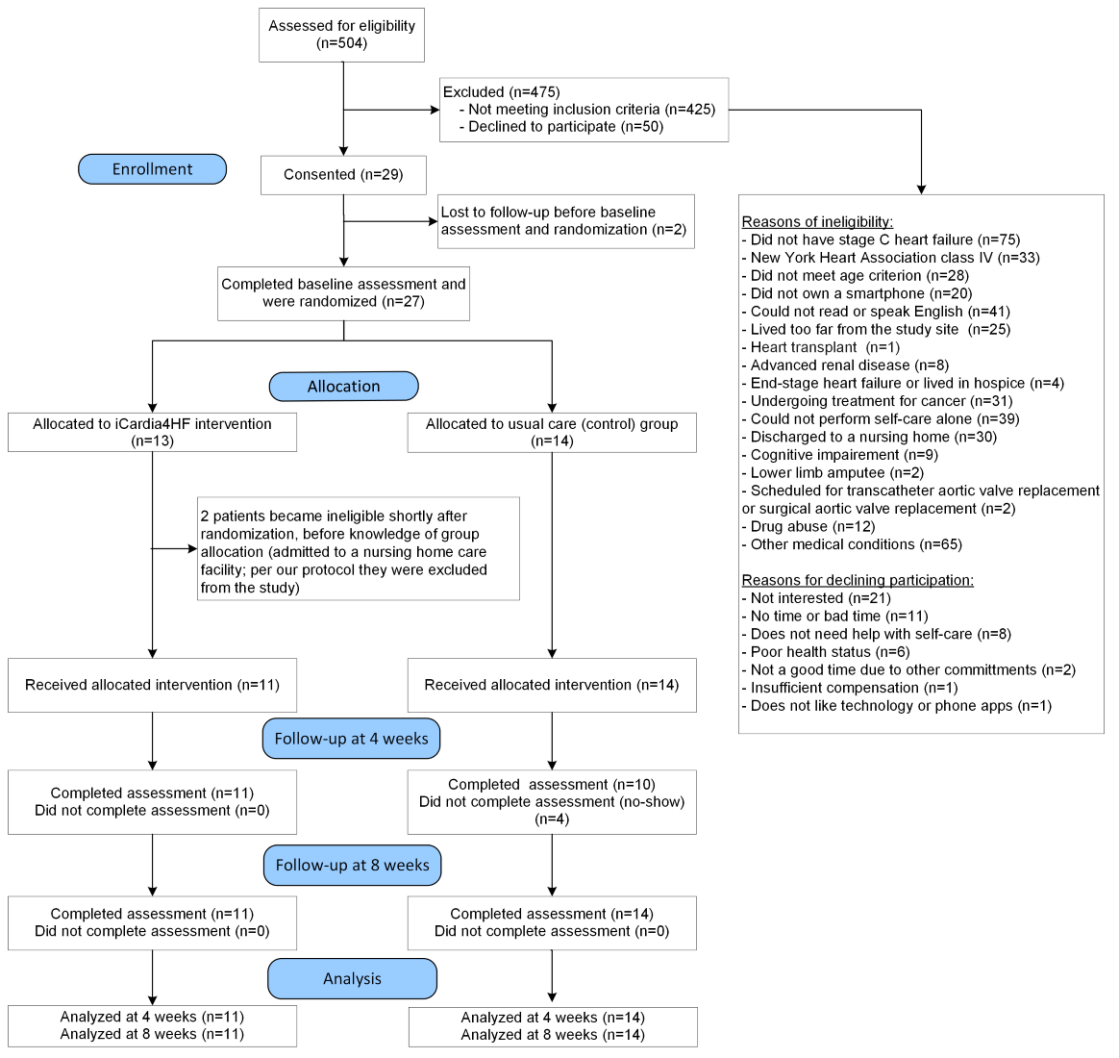
Trial profile diagram. The term “no-show” refers to patients not showing up to their scheduled study visit appointment.

Figure 2 shows the numbers of patients who were screened for eligibility, enrolled in the trial, randomly assigned to one of the two groups, received intended treatment, completed follow-up assessments, and included in the final analysis. A total of 504 patients were assessed for eligibility through chart review and clinic recruitment. Of those, 84.3% (n=425) were ineligible, and 9.9% (n=50) patients declined to participate or be screened for participation in the study, yielding a recruitment fraction of 5.8% (29/504); proportion of potential participants who enrolled in the trial). On average, we enrolled 3 patients per month during the 10-month recruitment period (from January 2019 to November 2019). Of the 29 participants enrolled in the study, 7% (2/29) were lost to follow-up before baseline assessment and randomization. The 27 participants who completed baseline assessment were randomized to the intervention (n=13, 45%) or the control group (n=14, 52%). Two participants allocated to the intervention group were excluded from the study because they were admitted to a nursing home and became ineligible before allocation of the mHealth app and devices.

Table 3 presents the characteristics of the 25 eligible patients who enrolled in and completed the study. The mean age of patients was 56 (range 41-72) years, and 44% (11/25) were female. Regarding self-identified race, 92% (23/25) were Black and 8% (2/25) were White. Baseline demographic and clinical characteristics were similar between the intervention and control groups.

**Table 3:** Baseline demographics and clinical characteristics.

Variables		Overall (n=25)	Intervention (n=11)	Control (n=14)
Age (y), mean (SD)		56 (8.3)	55.4 (9.17)	56.4 (7.8)
**Sex, n (%)**				
	Male	14 (56)	5 (46)	9 (64)
	Female	11 (44)	6 (55)	5 (36)
**Race, n (%)**				
	Black	23 (92)	10 (91)	13 (93)
	White	2 (8)	1 (9)	1 (7)
**Employment status, n (%)**				
	Working	9 (36)	3 (27)	6 (43)
	Unemployed	4 (16)	3 (27)	1 (7)
	Disabled	5 (20)	1 (9)	4 (29)
	Retired	7 (28)	4 (36)	3 (21)
**Marital status, n (%)**				
	Never married	12 (48)	5 (45)	7 (50)
	Married	7 (28)	4 (36)	3 (21)
	Divorced	2 (8)	0 (0)	2 (14)
	Widowed	4 (16)	2 (18)	2 (14)
**Living arrangement, n (%)**				
	Living alone	9 (36)	5 (46)	4 (29)
	Living with partner, spouse, or family	16 (64)	6 (55)	10 (71)
**Education, n (%)**				
	High school or less	7 (28)	4 (36)	3 (21)
	Some college (no degree)	8 (32)	4 (36)	4 (29)
	Community college or associate degree	4 (16)	2 (18)	2 (14)
	Undergraduate degree	5 (20)	1 (9)	4 (25)
	Graduate level degree	1 (4)	0 (0)	1 (7)
**Health insurance, n (%)**				
	Private insurance	10 (40)	3 (27)	7 (50)
	Medicaid	6 (24)	3 (27)	3 (21)
	Medicare	4 (16)	2 (18)	2 (14)
	Medicare and private insurance	3 (12)	2 (18)	1 (7)
	Managed Medicare (Medicare Advantage Plan)	1 (4)	1 (9)	0 (0)
	No insurance	1 (4)	0 (0)	1 (7)
**NYHA** ^a^ **class, n (%)**				
	I	6 (24)	3 (27)	3 (21)
	II	15 (60)	6 (55)	9 (64)
	III	4 (16)	2 (18)	2 (14)
**LVEF** ^b^ **(%)**				
	LVEF, mean (SD)	35.8 (12.4)	36.8 (11.5)	35.0 (13.4)
	≤40	15 (60)	6 (54.5)	9 (64.3)
	41-49	5 (20)	3 (27.3)	2 (14.3)
	≥50	5 (20)	2 (18.2)	2 (14.3)
**Blood pressure (mm Hg), mean (SD)**				
	Systolic	136.6 (20.14)	136.6 (20.14)	141.1 (22.4)
	Diastolic	82.4 (10.58)	82.4 (10.58)	83.9 (11.5)
Weight (kg), mean (SD)		110.8 (33.1)	109.2 (31.5)	112.1 (35.7)
BMI (kg/m^2^), mean (SD)		36.6 (10.1)	37.7 (11.21)	35.79 (9.54)
MoCA^c^ score, mean (SD)		25.92 (2.05)	26.7 (1.61)	25.29 (2.20)
**PHQ-9** ^d^ **score**				
	Score, mean (SD)	3.12 (3.5)	3.45 (3.4)	2.86 (3.8)
	0-4 (minimal or none), n (%)	17 (68)	7 (64)	10 (71)
	5-9 (mild), n (%)	6 (24)	3 (27)	3 (21)
	10-14 (moderate), n (%)	2 (8)	1 (9)	1 (7)

^a^NYHA: New York Heart Association.

^b^LVEF: left ventricular ejection fraction.

^c^MoCA: Montreal Cognitive Assessment.

^d^PHQ-9: Patient Health Questionnaire.

### Effects on HF Self-Care Scores

At 4 weeks, there was a clinically important difference (>8 points) between the 2 groups in maintenance (Cohen *d*=0.57, 95% CI –0.32 to 1.46) and self-care management (Cohen *d*=0.91, 95% CI –0.01 to 1.82), favoring the iCardia4HF intervention (Table 4). Symptom perception improved in both groups from baseline to 4 weeks, but there was no clinically relevant difference between the 2 groups (Cohen *d*=0, 95% CI –0.84 to 0.85).

At 8 weeks, participants in the intervention group reported, on average, greater improvement in self-care maintenance (Cohen *d*=0.19, 95% CI –0.65 to 1.02), symptom perception (Cohen *d*=0.33, 95% CI –0.51 to 1.17), and self-care management (Cohen *d*=0.25, 95% CI –0.55 to 1.04) than the control group (Table 4).

**Table 4:** Mean Self-Care Heart Failure Index (SCHFI) subscale scores and change over time by group.

HF^a^ self-care subscales		Intervention group, mean (SD)	Change in intervention group scores from baseline, mean (SD)	Control group, mean (SD)	Change in control group scores from baseline, mean (SD)	Between-group difference, Cohen *d* (95% CI)	*P* value
**Maintenance**							
	Baseline	69.55 (15.16)	—^b^	74.29 (17.82)	—	—	—
	4 weeks	75.68 (14.79)	6.14 (15.39)	70.90 (19.29)	–3.39 (18.16)	0.57 (–0.32 to 1.46)	.17
	8 weeks	75.91 (14.37)	6.36 (15.34)	78.04 (14.58)	3.75 (12.51)	0.19 (–0.65 to 1.02)	.65
**Symptom perception**							
	Baseline	79.80 (10.40)	—	75.79 (20.63)	—	—	—
	4 weeks	86.62 (12.96)	6.82 (10.04)	82.52 (18.79)	6.73 (22.61)	0 (–0.84 to 0.85)	.99
	8 weeks	87.63 (11.54)	7.83 (9.85)	80.16 (18.23)	4.37 (10.93)	0.33 (–0.51 to 1.17)	.41
**Self-care management**							
	Baseline	67.97 (10.93)	—	72.45 (16.98)	—	—	—
	4 weeks	73.16 (17.27)	5.19 (14.59)	64.80 (23.41)	–7.64 (14.07)	0.91 (–0.01 to 1.82)	.03
	8 weeks	73.16 (20.09)	5.19 (20.14)	73.64 (13.96)	1.19 (11.77)	0.25 (–0.59 to 1.09)	.56

^a^HF: heart failure.

^b^Not applicable.

### Effects on Intervention Target Measures (Health Beliefs, Self-Efficacy, and HF Knowledge)

Assessment of health beliefs focused on perceived benefits and perceived barriers of adherence to 3 important HF self-care behaviors: medication taking, low-sodium diet, and self-monitoring.

With respect to medication adherence, there were no important differences between the intervention and control group at 4 weeks, but there was a meaningful difference (*d*=0.63) in perceived benefits at 8 weeks, favoring the iCardia4HF intervention (Table 5).

With respect to diet, there were meaningful treatment effects with the iCardia4HF intervention in perceived benefits both at 4 and 8 weeks (Cohen *d*=0.85 and 0.55, respectively), but no meaningful differences between the intervention and control group in perceived barriers (Table 5).

In terms of self-monitoring, there were positive treatment effects in perceived benefits with the iCardia4HF intervention at 4 and 8 weeks (Cohen *d*=0.47 and 0.94, respectively), compared with the control group (Table 5).

There were clinically meaningful differences in self-efficacy scores between the intervention and control group both at 4 and 8 weeks, favoring the iCardia4HF intervention (Table 5). Compared to baseline, patients in the intervention group had an improvement in self-efficacy scores at both time points, while patient scores in the control group declined.

The iCardia4HF intervention had no effect on HF knowledge at 4 weeks but trended to have a small positive effect at 8 weeks (Cohen *d*=0.21).

**Table 5:** Mean intervention target scores (health beliefs, self-efficacy, and heart failure [HF] knowledge) and change over time and by group.

Subscales and time points		Intervention group, mean (SD)	Change in intervention group scores from baseline, mean (SD)	Control group, mean (SD)	Change in control group scores from baseline, mean (SD)	Between-group difference, Cohen *d* (95% CI)	*P* value
**Benefits of medication adherence**							
	Baseline	23.36 (3.41)	—^a^	24.24 (2.84)	—	—	—
	4 weeks	24.09 (3.56)	0.73 (3.69)	25.26 (4.35)	1.02 (3.24)	–0.09 (–0.96 to 0.78)	.84
	8 weeks	26.09 (3.30)	2.73 (3.38)	25.13 (3.53)	0.89 (2.51)	0.63 (–0.25 to 1.51)	.14
**Benefits of diet adherence**							
	Baseline	28.36 (4.32)	—	31.71 (3.15)	—	—	—
	4 weeks	30.36 (3.11)	2.00 (4.05)	30.10 (3.94)	–1.61 (4.53)	0.85 (–0.09 to 1.79)	.05
	8 weeks	31.00 (2.79)	2.64 (4.03)	32.00 (3.28)	0.29 (4.43)	0.55 (–0.30 to 1.40)	.17
**Benefits of self-monitoring**							
	Baseline	20.27 (4.82)	—	23.36 (3.65)	—	—	—
	4 weeks	22.36 (5.20)	2.09 (4.01)	23.53 (5.28)	0.17 (4.13)	0.47 (–0.41 to 1.35)	.27
	8 weeks	25.18 (3.16)	4.91 (4.35)	24.79 (3.40)	1.43 (3.11)	0.94 (0.06 to1.82)	.03
**Barriers of medication adherence**							
	Baseline	14.09 (3.02)	—	12.23 (4.09)	—	—	—
	4 weeks	15.64 (3.75)	1.55 (2.81)	13.62 (3.51)	1.39 (2.92)	0.04 (–0.87 to 0.95)	.90
	8 weeks	14.27 (4.76)	0.18 (4.40)	11.64 (3.20)	–0.59 (3.11)	0.21 (–0.65 to 1.06)	.63
**Barriers of diet adherence**							
	Baseline	11.91 (2.55)	—	12.50 (4.20)	—	—	—
	4 weks	11.27 (3.88)	–0.64 (2.29)	12.44 (4.81)	–0.06 (3.44)	–0.19 (–1.09 to 0.72)	.65
	8 weeks	11.64 (3.83)	–0.27 (2.05)	10.86 (3.37)	–1.64 (2.50)	0.59 (–0.26 to 1.44)	.13
**Barriers of self-monitoring adherence**							
	Baseline	21.91 (3.33)	—	26.57 (6.20)	—	—	—
	4 weeks	20.00 (4.92)	–1.91 (4.95)	23.42 (6.28)	–3.15 (6.00)	0.23 (–0.65 to 1.10)	.59
	8 weeks	19.09 (5.59)	–2.82 (5.15)	25.07 (6.97)	–1.50 (6.01)	–0.23 (–1.07 to 0.60)	.56
**Self-efficacy (SCSE** ^b^ **)**							
	Baseline	83.41 (10.97)	—	88.39 (11.25)	—	—	—
	4 weeks	89.09 (14.59)	5.68 (13.92)	87.29 (16.68)	–1.10 (12.20)	0.53 (–0.37 to 1.43)	.23
	8 weeks	90.91 (10.26)	7.50 (17.21)	85.89 (16.25)	–2.50 (12.33)	0.68 (–0.17 to 1.54)	.10
**HF-knowledge (DHFK** ^c^ **)**							
	Baseline	11.64 (1.50)	—	11.36 (2.44)	—	—	—
	4 weeks	11.54 (1.34)	–0.10 (2.05)	12.12 (1.67)	0.77 (2.07)	–0.42 (–1.27 to 0.43)	.30
	8 weeks	12.09 (1.22)	0.45 (1.97)	11.36 (1.98)	0.00 (2.25)	0.21 (–0.62 to 1.05)	.59

^a^Not applicable.

^b^SCSE: Self-Care Self-Efficacy scale.

^c^DHFK: Dutch Heart Failure Knowledge Scale.

### Effects on Health Status Scores

With respect to health status (Table 6), patients in the intervention group had moderate effect size improvements that were clinically meaningful (>5 points difference), both at 4 weeks (Cohen *d*=0.54) and 8 weeks (Cohen *d*=0.46). Improvements in the intervention arm were mainly driven by positive changes in the physical limitation, symptom frequency, and social limitation domains.

**Table 6:** Change in Kansas City Cardiomyopathy Questionnaire 12-item (KCCQ-12) subscale scores over time by group.

Health status (KCCQ-12)		Intervention group, mean (SD)	Change in intervention group scores from baseline, mean (SD)	Control group, mean (SD)	Change in control group scores from baseline, mean (SD)	Between-group difference, Cohen *d* (95% CI)	*P* value
**Physical limitation**							
	Baseline	62.50 (36.18)	—^a^	71.43 (26.90)	—	—	—
	4 weeks	82.58 (21.56)	20.08 (27.44)	69.79 (29.31)	–1.64 (36.51)	0.66 (–0.21 to 1.54)	.10
	8 weeks	87.88 (21.53)	25.38 (22.86)	83.93 (20.73)	12.50 (28.50)	0.49 (–0.35 to 1.34)	.21
**Symptom frequency**							
	Baseline	68.56 (33.14)	—	77.08 (29.19)	—	—	—
	4 weeks	74.81 (28.87)	6.25 (36.38)	82.70 (24.35)	5.62 (28.90)	0.02 (–0.84 to 0.88)	.96
	8 weeks	80.49 (20.23)	11.93 (29.18)	80.21 (28.16)	3.13 (26.86)	0.32 (–0.52 to 1.15)	.44
**Quality of life**							
	Baseline	57.95 (33.67)	—	57.14 (26.27)	—	—	—
	4 weeks	65.91 (30.66)	7.95 (31.76)	60.67 (25.21)	3.53 (20.99)	0.18 (–0.69 to 1.04)	.70
	8 weeks	69.32 (28.70)	11.36 (24.66)	68.75 (23.39)	11.61 (20.49)	–0.01 (–0.84 to 0.82)	.98
**Social limitation**							
	Baseline	66.86 (42.89)	—	82.74 (17.44)	—	—	—
	4 weeks	81.82 (27.08)	14.96 (31.34)	77.29 (22.21)	–5.45 (25.71)	0.72 (–0.17 to 1.62)	.10
	8 weeks	79.92 (26.80)	13.07 (31.07)	77.38 (30.21)	–5.36 (32.46)	0.58 (–0.28 to 1.44)	.16
**Overall score**							
	Baseline	63.67 (33.34)	—	72.10 (19.93)	—	—	—
	4 weeks	76.28 (23.35)	12.61 (23.82)	72.22 (20.79)	0.12 (22.80)	0.54 (–0.33 to 1.41)	.19
	8 weeks	79.40 (21.80)	15.74 (22.93)	77.57 (23.04)	5.47 (21.81)	0.46 (–0.38 to 1.30)	.26

^a^Not applicable.

### Self-Monitoring Adherence

Daily weighing and BP monitoring adherence over 8 weeks was 86.4% (SD 11.16%) and 86.22% (SD 10.35%), respectively, among patients allocated to the iCardia4HF intervention. Daily self-monitoring of weight and BP decreased by –0.2% (t_78_=–0.32; *P*=.75) and –0.9% (t_78_=–1.25; *P*=.21) each week during the intervention period, but the decline was not statistically significant (Table 7). From baseline to 4 weeks, mean daily weighing adherence was 85.5% (SD 20.8%), and mean daily BP monitoring adherence was 86.6% (SD 20.3%). From week 4 to week 8, mean daily weighing adherence was 87.2% (SD 10.8%), and daily BP monitoring adherence was 85.8% (SD 11.9%).

**Table 7:** Linear mixed-effects analyses of daily weighing and blood pressure (BP) monitoring adherence over time (intervention group).

Outcome	Variable	Mean (SE)	*t* test (*df*)	*P* value
BP	Intercept	92.08 (4.90)	18.76 (78)	<.001
BP	Slope	–0.95 (0.77)	–1.25 (78)	.21
Weight	Intercept	88.74 (5.19)	17.10 (78)	<.001
Weight	Slope	–0.26 (0.83)	–0.32 (78)	.75

### Effects on Physical Activity

As shown in Table 8, the Fitbit activity tracker and Fitbit mobile app used for promotion and self-monitoring of physical activity did not have a clinically meaningful effect on the intervention participants’ trajectory of daily steps, MVPA, or sedentary minutes over the 8-week intervention period. Overall, participants averaged 5480 (SD 3904) steps per day and 22.20 (SD 18.71) MPVA minutes per day during the 8-week intervention period. From baseline to 4 weeks, participants averaged 5341.49 (SD 3923.18) steps per day and 19.78 (SD 21.88) MVPA minutes per day. From 4 to 8 weeks, intervention participants averaged 5619.20 (SD 4015.03) steps per day and 24.62 (SD 20.45) MVPA minutes per day. On average, intervention participants wore their Fitbit for 1031 (SD 421.2) minutes per day during the 8-week intervention period. Mean adherence to wearing the Fitbit for ≥600 minutes per day (as recommended at study orientation) was 85.3% (SD 23.12%). Wear-time slightly declined over the 8-week period by an average of 36 minutes per day (t_76_=–4.46; *P*<.001).

**Table 8:** Linear mixed-effects analyses of physical activity and Fitbit wear-time over time (intervention group).

Outcome	Variable	Values, mean (SE)	*t* test (*df*)	*P* value
Daily steps	Intercept	5085.50 (1281.37)	3.97 (65)	<.001
Daily steps	Slope	25.70 (72.66)	0.35 (65)	.72
MVPA^a^ min/d	Intercept	17.35 (6.93)	2.50 (65)	.01
MVPA min/d	Slope	0.687 (0.76)	0.90 (65)	.37
Sedentary min/d	Intercept	637.01 (38.88)	16.38 (65)	<.001
Sedentary min/d	Slope	1.98 (3.28)	0.60 (65)	.54
Fitbit wear-time min/d	Intercept	1191.90	11.82 (76)	<.001
Fitbit wear-time min/d	Slope	–36.12	–4.46 (76)	<.001

^a^MVPA: moderate-to-vigorous physical activity.

### Acceptability of the Intervention

As shown in Table 9, most patients perceived the intervention to be useful in supporting HF self-care (mean 2.94 on a 3-point Likert scale, SD 0.07). During exit interviews, patients said that they found the intervention very engaging and that the mobile apps and devices empowered them to perform daily self-monitoring of weight, symptoms, and physical activity. Also, participants said that the text messages provided positive reinforcement and helped them become more informed about HF and self-care. Importantly, most patients reported that they felt more confident in managing their condition, and the data in the apps allowed them to have more informed discussions with their care providers during follow-up appointments. Most patients found the apps and devices easy to use (mean 2.97, SD 0.08) and positively commented on the ability of devices to automatically transfer data to the mobile apps on their phone via Bluetooth, thus eliminating any need for manual data entry. Overall, patients claimed to be very satisfied with the intervention components (mean 2.93, SD 0.14) and had a firm intention of continuing to use the apps and devices of the iCardia4HF intervention after the end of the study (mean 3, SD 0).

**Table 9:** Acceptance of the iCardia4HF intervention.

Subscales and measures		Scores, mean (SD)
**Perceived usefulness (overall score)**		2.94 (0.07)
	I have maintained or improved my health by using the mobile app and smart devices of the iCardia4HF intervention	2.90 (.32)
	I am more informed about my heart failure by using the mobile app and smart devices	3 (0)
	My knowledge about self-managing my health has improved	3 (0)
	I feel more confident taking care of my health	3 (0)
	I am more autonomous in monitoring my vital signs and heart failure symptoms	3 (0)
	I feel less anxious about my health	2.80 (0.42)
	I have more informed discussions with my doctor about my heart failure based on the data I collect from the mobile app and smart devices	2.90 (0.32)
**Ease of use (overall score)**		2.97 (0.08)
	I found it easy to use the mobile app and wearable device(s) for self-monitoring my heart failure	3 (0)
	I found the mobile app and wearable/smart device(s) user-friendly	3 (0)
	Learning how to use the mobile app and wearable/smart device(s) to self-manage my heart failure was easy for me	2.90 (0.32)
	The information provided/stored in the mobile app(s) was easy to understand and interpret	3 (0)
	Interacting with the mobile app and wearable/smart devices was clear and understandable	3 (0)
**User satisfaction (overall score)**		2.93 (0.14)
	I am satisfied with the use of the mobile app and the wearable/smart devices for self-monitoring	3 (0)
	I am pleased with the use of the wearable/smart devices	3 (0)
	I am delighted with the use my wearable/smart devices	2.80 (0.42)
**Confirmation of initial expectations (overall score)**		2.90 (0.23)
	My initial expectations concerning the use of the heart failure mobile app and wearable/smart devices have been confirmed so far	2.70 (0.68)
	Using the heart failure mobile app and wearable/smart devices turned out to be easier that I first thought	3 (0)
	There are more benefits to using the heart failure mobile app and wearable/smart devices than I first thought	3 (0)
**Intention to continue using (overall score)**		3 (0)
	I have every intention of continuing to use the mobile app and wearable/smart devices in the future	3 (0)
	I will continue to use the mobile app and wearable/smart devices to monitor different aspects of my health	3 (0)
	I have no intention of stopping to use the heart failure mobile app and wearable/smart devices in the future	3 (0)

### Adverse Events

During the study follow-up period of 8 weeks, there were 6 hospitalizations: 4 in the intervention group (1/11 patients, 9% experienced an event) and 2 in the control group (1/14 patients, 7% experienced an event). There were also 4 emergency room visits: 2 in the intervention group (2/11 patients, 18% experienced an event) and 2 in the control group (1/14 patients, 7% experienced an event). In terms of HF-related hospitalizations, there was 1 HF readmission in the intervention group (1/11 patients, 9% experienced an event) and none in the control group. There were no deaths during the follow-up period. There were no serious or other adverse events associated with the intervention or study procedures.

## Discussion

### Principal Findings and Interpretation

In this phase 1 randomized controlled trial, we examined the feasibility, acceptability, safety, and preliminary efficacy of a patient-centered mHealth intervention (iCardia4HF) on HF self-care and other health outcomes in a predominantly racial and ethnic minority population of middle-aged and older adult patients with HF. The iCardia4HF intervention integrates multiple consumer-grade mHealth apps and devices (MyApps) with a program of individually tailored text messages (Text4HF) targeting HF self-care and modifiable behavioral factors affecting HF self-care. Overall, our results demonstrated that iCardia4HF is a potentially feasible, acceptable, and safe intervention that warrants further exploration in a phase 2 clinical trial to determine its efficacy.

The results of our recruitment efforts provide important information that can be used in the planning of a phase 2 trial and other future mHealth intervention studies in midlife and older adult patients with HF. The numbers of patients eligible for the study were much lower than we originally anticipated and required a significantly larger pool of patients with HF to be screened during the recruitment period. The data show that approximately 16 patients needed to be screened for every patient enrolled (5.8% recruitment fraction). Although low, this rate is consistent with rates reported in other studies of patients with HF [90]. For example, the Better Effectiveness After Transition–Heart Failure study [91], one of the largest RCTs of remote patient telemonitoring in an HF population aged ≥50 years in the United States (n=1437 patients with HF, 664/1437, 46.2% female, and 316/1437, 22% African American), had a recruitment fraction of 4.7%. The study assessed 30,844 patients for eligibility and enrolled 1437 patients. Approximately 92.32% (28,476/30,844) of people screened did not meet the inclusion criteria. These numbers highlight the challenge of conducting RCTs in patients with serious chronic diseases, such as HF, who often have multiple comorbidities, functional disabilities, or other impairments that affect eligibility and may limit their ability to participate in research [90]. In our study, comorbidities (eg, cancer and severe or end-stage chronic kidney disease) and functional or cognitive impairments (eg, inability to walk, dementia, and Alzheimer disease) accounted for approximately 45% of exclusions. Future studies might consider modifying the eligibility criteria to enable testing of the iCardia4HF intervention in a larger and more diverse population with HF. Taken collectively, these results emphasize the need to carefully develop sound recruitment strategies before a study begins and devote sufficient financial and personnel resources to meet recruitment needs.

Despite the lower than anticipated recruitment rate, we achieved a high degree of participant retention and completion of study visits (85% at 4 weeks and 100% at 8 weeks). We also observed high rates of adherence (>80%) to wearing the Fitbit activity tracker and using the mHealth apps and devices daily for self-monitoring of vital signs and HF symptoms. Quantitative ratings from study participants allocated to the intervention (100% response rate) showed high levels of perceived usefulness, ease of use, and intention to continue using the technology for HF self-care. Patients were also satisfied with the text messages (Text4HF) and said that they provided them with positive reinforcement. These findings are consistent with previous studies and reviews on technology adoption, which report that older adults are more likely to adopt and consistently use a new technology if it is perceived as useful (ie, addresses an important need) and is easy to use [92-94]. The functional and cognitive changes that come with aging, such as poorer vision, cognitive decline, memory loss, decreased dexterity, and sensory impairments, make learning and using mHealth apps and wearable devices more challenging for older adults [94]. Hence, it is important for mHealth technologies to meet a series of end user requirements, such as user-friendly designs, limited manual data entry, easy-to-interpret visualizations, and appropriate user support [95,96]. Qualitative feedback during exit interviews illuminated that the salient features of our mHealth intervention supporting technology acceptance and satisfaction were (1) the automated capturing and syncing of the data, which eliminated the need for manual data entry into the mHealth apps; (2) the overall user-friendliness and ease of use of the intervention apps and devices; (3) the helpful content and positive reinforcement that the text messages provided; and (4) the feedback received from the intervention apps in the form of notifications and data visualizations. Another factor that most likely played a key role in the acceptance and use of the intervention technology, although not mentioned in the exit interviews, was the training and technical support participants received during the study. Participants received one-on-one instruction at baseline on how to use each mHealth app and device, including clear and simplified end user guides that our team developed for this study. They were also provided with a number they could call during office hours to receive technical support from study staff for individual problems with the devices. Previous research has consistently shown that with sufficient training and support, older adults are significantly more likely to learn and use new technologies, even if they have limited prior experience [97-100]; this is because they may need more time and tailored instruction to overcome potential barriers to adoption. Other factors that may have played a positive role in the acceptance and use of the intervention technology relate to the characteristics of our study sample. Specifically, the relatively younger mean age (56 years) of our HF population, which is reflective of the earlier age of HF onset in Black or African American people [62], and patient selection criteria of our study (eg, smartphone ownership, ability to independently perform self-care, and no major cognitive decline). Future studies and pragmatic trials are needed to determine whether older (aged ≥65 years) and more diverse populations with HF will be as willing to adopt and consistently use our intervention technologies over a longer period.

With respect to the preliminary efficacy of the intervention, iCardia4HF trended to improve HF self-care as indicated by the change in SCHFI scores from baseline. Even though the CIs were wide and included the null, there was a consistently positive effect on all 3 HF self-care domains (maintenance, symptom perception, and self-care management) in the intervention group. The between-group Cohen *d* effect sizes of 0.19, 0.33, and 0.25 for the 3 HF self-care subscales of *maintenance*, *symptom perception*, and *self-care management* at 8 weeks, respectively, qualify as a “probably positive” finding, according to the various scenarios prespecified in the study protocol about the possible intervention effect [68]. The SCHFI subscale scores observed in our study were higher at baseline compared with those reported in other studies in the literature [101-104], which means that, on average, many study participants were already performing adequate HF self-care when enrolled in our study based on self-report. This may have made it more difficult to demonstrate larger changes in HF self-care between the intervention and control groups. To address this issue, in the phase 2 trial of iCardia4HF [68], we are using SCHFI in the screening process to identify patients who are performing insufficient self-care and are more likely to benefit from the iCardia4HF intervention [105]. This approach has been used in other trials [106]. Given the small sample of our study, we found no significant correlations between HF self-care and patient demographic or clinical characteristics (eg, age, sex, living alone, education, employment, LVEF, and NYHA). Future research should further explore the role of these factors as potential confounders or mediators of HF self-care [107].

Compared with usual care, the intervention trended toward improvement in self-reported health status (physical limitation, symptom frequency, and social limitation) as assessed with the KCCQ-12. The greatest improvements in terms of Cohen *d* values were observed in the intervention target variables of self-efficacy (0.68) and health beliefs about HF self-care, especially perceived benefits of medication adherence (0.63) and self-monitoring adherence (0.94). A possible explanation for this finding is that these measures, in addition to the mHealth apps and devices (MyApps), were directly targeted by the tailored text messaging component (Text4HF), which may have had an additive effect. However, this finding requires further exploration. In a new clinical trial that we are conducting with funding from the National Institute of Heart and Lung Blood Institute (1 R01 HL168376-01), we are using a 2×2 factorial randomized trial design to determine the independent and synergistic effects of the 2 iCardia4HF intervention components (MyApps and Text4HF).

Physical activity (steps and MVPA) measured with the Fitbit activity tracker in the intervention group only did not improve over time. A possible explanation for this finding, besides the short duration of the study, is the lack of specific physical activity goals pertaining to the 2 measures of interest: steps and MVPA. A recent systematic review and meta-analysis of Fitbit-based physical activity interventions showed that goal setting is more effective than Fitbit alone [51]. iCardia4HF could potentially be strengthened in this area by incorporating weekly physical activity goal setting for either steps or MVPA to gradually increase participants’ physical activity as recommended by existing guidelines [1,108].

### Comparison With Other Studies

In a recent systematic review and meta-analysis that we conducted and published [29], we identified 16 RCTs (n=4389 patients) of mHealth interventions for patients with HF. Most trials (12/16, 75%) evaluated the efficacy of RPM interventions (also known as telemonitoring) that included daily transmission of patient-generated health data to a clinical care team for review and delivery of actionable feedback based on the incoming data [29]. Fewer trials (n=4) tested the efficacy of stand-alone, patient-centered interventions (without RPM) targeting HF self-care through the use of mobile technologies. Of these 4 trials, 3 (75%) tested the efficacy of a single self-care support mobile app [102,109,110], while one trial [111] tested the efficacy of HF self-care text messages compared to usual care. Another RCT published after our systematic review tested a tablet-based mobile app designed to support HF patients’ self-care at home [112]. Sample size in the 5 trials of mHealth interventions without RPM ranged from 18 patients to 767 patients, and follow-up duration ranged from 30 days to 6 months.

Daily adherence to using the app was reported to be high (>80%) among intervention participants in 2 studies [109,112], while in another study [102], patient engagement with the app and study completion were affected by challenges faced with using a chest-strap Bluetooth device (BioHarness-3) for vital signs and exercise monitoring. Only 72% (13/18) of participants completed the 30-day follow-up, and 43% (4/9) accessed the app daily.

HF self-care in the 5 RCTs was assessed using different instruments. A total of 2 studies used an older SCHFI version (version 6.2) [102,110], another 2 studies used different versions of the European Heart Failure Self-care Behavior Scale (EHFScB-12 and EHFScB-9) [109,112], and 1 study used generic items about self-care (not previously validated). Consequently, it is difficult to directly compare the results of these studies with our effect sizes and 95% CI. The 2 trials that used EHFScB found statistically significant improvements in the overall self-care score with the intervention, while results in the other 2 trials that used SCFHI were inconsistent. Athilingam et al [102] found significant improvements in the self-care management and self-care confidence subscales, while Dorsch et al [110] found no differences between the intervention and control groups in the SCHFI subscales.

Contrary to our intervention, the mobile apps used in these studies were not commercially available. They were developed internally by the research team and with input from health care providers (eg, cardiologists, HF nurses, and dieticians). In terms of HF self-care supported features, all 4 apps prompted active daily self-monitoring of weight and HF symptoms, included an education module on HF, and generated automated alerts of possible decompensated HF using an algorithm based on predetermined risk criteria or scores. The latter was a feature that our intervention did not have. Two apps [102,112] included a medication tracker feature and supported physical activity and exercise monitoring, and 1 app supported daily salt intake and dietary records [112]. Only 1 app supported wireless transfer of data from external devices (eg, weight scale) to the app. The other 3 apps required users to manually enter their vital signs recorded from external devices into the app (eg, weight, heart rate, BP, and steps). This is primarily due to the difficulty of integrating third-party devices with a custom-made app.

One of the 5 RCTs was a large trial (n=767) conducted in China [111] that investigated the efficacy of an SMS text messaging intervention on the composite end point of death- or readmission-free survival and the secondary outcome of self-care behavior compared with usual care over 180 days. Participants were recently discharged patients with ADHF (mean age 61, SD 15 years; 334/767, 43.5% female and 525/767, 68.4% NYHA class 3). Patients in the SMS group, as well as their caregivers, received educational and reminder text messages from a platform operated by research nurses. The educational text messages were condensed SMS about HF knowledge (eg, symptoms of HF decompensation), while the reminder text messages focused on self-care adherence (eg, taking medicine or weighing). Results showed that event-free survival was better in the SMS group when compared with the control group (odds ratio 0.819, 95% CI 0.677-0.991). In terms of self-care, patients in the SMS group reported better medication compliance (*P*=.03) and water restriction (*P*=.046) than the control group, but there were no significant differences in daily weighing adherence, restricted sodium diet, and physical activity. The latter finding might be attributed to how self-care was assessed and to what extent the text messaging program targeted these self-care behaviors (daily weighing, low-sodium diet, and physical activity). Contrary to our study, self-care adherence was not assessed with digital devices or a validated instrument, and messages in this trial were not tailored to study participants. Text messages had a limited scope compared with our Text4HF program, which, in addition to HF knowledge and self-care adherence, focused on changing health beliefs (perceived benefits and barriers of HF self-care adherence) and increasing self-efficacy.

Previous research has shown that health communication interventions that succeed in targeting specific content areas and making information relevant to their intended audience are more effective than those that do not. In a pilot single-arm prospective study (n=15) of a text messaging intervention in a largely African American population with ADHF, Nundy et al [103] found that specific SCHFI items that improved over time were generally those directly targeted by the text messages [103]. Other self-care items, such as physical activity and exercise that were not targeted by the intervention did not improve. This is consistent with the findings of our study and highlights the importance of message tailoring and careful selection of intervention, target variables and behaviors. Despite differences in the content and structure of the messaging intervention compared with our study, the positive results observed in the Chen et al [111] trial highlight the potential contribution of text messaging as a stand-alone or adjunct intervention toward improving HF self-care adherence and event-free survival in patients with HF, especially when postdischarge follow-up may not be readily available due to geographical barriers and care provider shortages.

### Study Strengths

This study has several strengths. First, we used a rigorous RCT design with allocation concealment and blinded outcome assessments at all time points to minimize selection bias and detection bias [113]. Second, we used an intention-to-treat analysis to minimize attrition bias [113]. Third, our intervention involved the use of mHealth apps and devices (eg, Fitbit and Withings) that are internationally known and widely available in different countries. This choice increases the potential for scalability and broader impact if the intervention is found to be effective in future studies. Fourth, we had a high representation of minority and female patients. African Americans and women are disproportionally studied in HF clinical trials compared with HF prevalence [5,114]. Fifth, we had a high rate of patient retention and compliance associated with the intervention. This allowed for optimal testing and delivery of our intervention. Sixth, the use of a previously validated digital health platform (iCardia) ensured reliable and consistent data collection from the mHealth apps and devices, including delivery of the text messaging intervention (intervention fidelity).

### Study Limitations

Though several promising results were produced, this study is not without limitations. Two important limitations are the small sample size and short follow-up duration. This study was not powered to detect statistically significant differences between the 2 groups, and therefore, effect sizes should be interpreted with caution, taking into consideration the uncertainty of the estimates as reflected by the 95% CI. A larger and longer-duration study is needed, including a maintenance period to assess the sustainability effects of the intervention. Another limitation was that our primary efficacy outcome of HF self-care was a self-reported measure, which is known to be influenced by detection bias when patients are unblinded to treatment allocation. Although we used objective measures and data from the apps and devices to assess self-monitoring adherence, these behaviors do not cover the entire spectrum of HF self-care activities that patients need to perform to maintain physiological stability. In the phase 2 trial of the iCardia4HF intervention [68], we incorporate additional methods and tools to objectively assess other important self-care behaviors, such as medication adherence (eg, pill monitoring bottles), low-sodium diet (eg, urine sodium), and completion of follow-up appointments (eg, EMR). Our intervention was not designed for people who have end-stage renal disease (exclusion criterion in this study), and therefore, our results may not be generalizable to this population. Also, the compensation of enrolled patients for their participation in the study, along with the possibility of the Hawthorn effect (modification of behavior in response to awareness of being monitored), may have influenced the results. Finally, the health belief scales that guided the text messaging intervention were developed many years ago and have not been updated since. Therefore, many items focused on adherence to water pills, a low-sodium diet, and weight monitoring. In the future, we plan to include additional intervention target variables and expand our database of behavior change text messages to other healthy lifestyle behaviors, such as heart-healthy diet, guideline-directed medical therapy, smoking cessation, alcohol cessation, physical activity, and exercise. The methodological limitations described earlier (eg, sample size, short intervention, and follow-up duration) are reflective of a phase 1 feasibility trial.

### Implications for Future Research and Practice

This study contributes importantly to the limited body of literature on stand-alone mHealth interventions for patients with HF. Furthermore, it directly responds to an American Heart Association scientific statement on the current science of consumer mHealth technologies for CVD prevention [33], which recommended that future intervention studies include commercially available mHealth apps and devices to determine their safety and efficacy on health outcomes and how to best incorporate these technologies (once proven) into a broader collaborative model of care.

To our knowledge, this is the first RCT of a patient-centered mHealth intervention (without RPM) that tested the feasibility, safety, and preliminary efficacy of multiple commercial mHealth apps and devices in patients with HF. Previous studies and efforts have mainly focused on developing a single mobile app that supports most, if not all, functions of HF self-care. This is not only difficult to achieve given the fragmented nature of the current mHealth ecosystem, but also fails to consider that people tend to use multiple “best of breed” mobile apps in short bursts to support their daily activities. iCardia4HF is a departure from previous models and studies. Rather than relying on a single app, iCardia4HF provided patients with an integrated set of popular mHealth apps and devices (MyApps), each of which specialized in different preventative behaviors covering the main areas of HF self-care and supplemented these with a program of individually tailored text messages. The combination of smartphones and low-cost mHealth technologies that are commercially available makes the proposed intervention portable to other settings and creates exciting opportunities for scalability and broader impact. Text messaging is a proven, established technology that is both inexpensive and one of the most widely adopted mobile phone functions among adults. Unfortunately, text messaging for HF has been overlooked in favor of more advanced and “shiny” RPM technologies, including mobile apps and wearable sensor devices [115]. However, there is much to be learned from text message–delivered behavioral interventions. In our study, intervention patients indicated that text messages provided them with useful information, increased perceived support, enhanced motivation to engage in better HF self-care, and provided useful prompts for healthy behavior change. Additional trials are urgently needed to determine the efficacy of text messaging both as a stand-alone and as an adjunct intervention and to build a more robust evidence base for patients with HF.

### Conclusions

The iCardia4HF intervention proved to be feasible, acceptable, and safe with no evidence of increased adverse effects on HF symptoms or acute health care use (hospital readmissions and emergency visits). These results, along with the positive effects observed in HF self-care behaviors and other health outcomes, provide good evidence that a full-scale RCT to more definitively examine the efficacy of the iCardia4HF intervention in patients with HF is warranted.

## Appendix

Multimedia Appendix 1 CONSORT-eHEALTH (Consolidated Standards of Reporting Trials of Electronic and Mobile Health Applications and Online Telehealth) checklist (V 1.6.1).

## Abbreviations

ADHF: acute decompensated heart failure
BP: blood pressure
EF: ejection fraction
EHFScB: European Heart Failure Self-care Behavior Scale
HF: heart failure
HFmEF: heart failure with mild ejection fraction
HFpEF: heart failure with preserved ejection fraction
HFrEF: heart failure with reduced ejection fraction
HIPAA: Health Insurance Portability and Accountability Act
KCCQ-12: Kansas City Cardiomyopathy Questionnaire 12-item
LVEF: left ventricular ejection fraction
mHealth: mobile health
MVPA: moderate-to-vigorous physical activity
NYHA: New York Heart Association
RPM: remote patient monitoring
SCHFI: Self-Care Heart Failure Index
UI Health: University of Illinois Hospital and Health Sciences System
